# The Circadian Syndrome Is a Significant and Stronger Predictor for Cardiovascular Disease than the Metabolic Syndrome—The NHANES Survey during 2005–2016

**DOI:** 10.3390/nu14245317

**Published:** 2022-12-14

**Authors:** Zumin Shi, Jaakko Tuomilehto, Noga Kronfeld-Schor, George Alberti, Naftali Stern, Assam El-Osta, Zhonglin Chai, Carmel Bilu, Haim Einat, Paul Zimmet

**Affiliations:** 1 Human Nutrition Department, College of Health Sciences, QU Health, Qatar University, Doha 2713, Qatar; 2 Department of Public Health, University of Helsinki, 00014 Helsinki, Finland; 3 Population Health Unit, Finnish Institute for Health and Welfare, 00280 Helsinki, Finland; 4 Saudi Diabetes Research Group, King Abdulaziz University, Jeddah 21589, Saudi Arabia; 5 School of Zoology, Tel Aviv University, Tel Aviv 6997801, Israel; 6 Department of Endocrinology and Metabolism, Imperial College London, London SW7 2BX, UK; 7 Sagol Center for Epigenetics of Aging and Metabolism, Tel Aviv Medical Center, Tel Aviv 6997801, Israel; 8 Sackler Faculty of Medicine and Sagol School of Neuroscience, Tel Aviv University, Tel Aviv 6997801, Israel; 9 Department of Diabetes, Central Clinical School, Monash University, Melbourne, VIC 3004, Australia; 10 Hong Kong Institute of Diabetes and Obesity, The Chinese University of Hong Kong, Hong Kong, China; 11 School of Behavioural Sciences, Tel Aviv-Yaffo Academic College, Tel Aviv 6818211, Israel

**Keywords:** circadian syndrome, metabolic syndrome, cardiovascular disease, adults, NHANES

## Abstract

The study aimed to compare the predictive value of the Circadian Syndrome (CircS) and Metabolic Syndrome (MetS) for cardiovascular disease (CVD). We used data of 12,156 adults aged ≥20 years who attended National Health and Nutrition Examination Survey (NHANES) 2005–2016. Mortality was obtained from the registry updated to 2019. The CircS was defined based on components of the MetS, in addition to short sleep and depression. Both the MetS and CircS were directly associated with self-reported history of CVD. The odds ratios for prevalent CVD associated with the CircS and MetS, respectively, were 2.92 (95% confidence interval (CI) 2.21–3.86) and 3.20 (2.38–4.30) in men, and 3.27 (2.34–4.59) and 3.04 (2.15–4.30) in women. The CircS had a better predictive power for prevalent CVD than that of MetS, as indicated by the higher positive predictive value (PPV); in men, the PPV for prevalent CVD with CircS was 23.1% and with MetS 20.9%, and in women these were 17.9% vs. 16.4%, respectively. However, the PPV of the CircS and MetS did not differ for the CVD mortality prediction. Women with CircS alone had a higher risk for both prevalent CVD and CVD mortality than those with MetS alone. In conclusion, the CircS is a significant and stronger predictor for CVD than the MetS in US adults.

## 1. Introduction

Globally, the burden of cardiovascular disease (CVD), as well as of some of its modifiable risk factors, continues to increase. Based on the Global Burden of Disease (GBD) Study 2019, the number of CVD cases doubled from 271 million in 1990 to 523 million in 2019 [1]. Identifying risk factors for CVD is vital for its prevention. The concept of the Metabolic Syndrome (MetS) has been proposed as a cluster of CVD risk factors that covers multiple components, including raised blood pressure, dyslipidaemia (raised blood triglycerides and low high-density lipoprotein cholesterol), elevated fasting blood glucose and central obesity [2]. The link between the MetS and CVD is well-established [3,4,5]. The MetS is becoming a global epidemic affecting both high- and low-income countries [6]. Based on the National Health and Nutrition Examination Survey (NHANES) 2011–2016 data, the weighted prevalence of the MetS was 34.7% in the US population [7]. Various mechanisms including insulin resistance [8], central obesity-related inflammation [9] and genetics [10], have been proposed as mechanisms of the MetS contributing to the risk of CVD, but there is a lack of consensus on this. Furthermore, there may be additional factors which can significantly contribute to the risk of CVD and should be considered in order to better predict the risk of CVD in those with metabolic disorders.

Indeed, in addition to all the above-mentioned mechanisms, circadian dysfunction has been hypothesized to be an important underlying etiological factor for the MetS [11]. Therefore, the concept of the Circadian Syndrome (CircS) is proposed based on the findings that many common chronic disorders such as obesity, hypertension, dyslipidaemia, type 2 diabetes, depression, sleep disorder and non-alcoholic fatty liver disease (NAFLD) have a strong link with circadian disruption. Modern lifestyles (e.g., sleep disorders, meal skipping, high-fat diet, sedentary behaviors, and shift work) as well as our living environment (e.g., exposure to artificial light at night and too little light indoors during daytime) can cause circadian rhythm disruption and affect health outcomes [11]. Therefore, it has been proposed that the CircS in addition to the MetS should be considered as a novel CVD risk cluster [11]. As opposed to CircS, sleep disorder and depression are not part of MetS.

Previous studies on circadian disruption and chronic disease have mainly focused on specific behaviors, such as shift work, irregular or short sleep, and artificial light exposure. Only two recent studies use the concept of CircS to categorize individuals and examine its association with health outcomes [12,13]. 

We have previously reported that the CircS is a better predictor for CVD than the MetS in the Chinese population [12]. However, it is unknown whether this finding is also valid for other populations. Using data from the NHANES 2005–2016, we aimed to answer the question.

## 2. Materials and Methods

NHANES is a cross-sectional survey run by the US Center for Disease Control and Prevention. It uses a multistage probability sampling technique to select a representative sample of the non-institutionalized population in the USA. A variety of methods were used in the data collection, including interviews (face-to-face or phone), questionnaires, laboratory testing and physical examination. Interviews were conducted in the home of each participant, while mobile examination centers were used to conduct the physical examination and collect blood samples [14]. The National Center for Health Statistics Institutional Ethics Review Board approved the study, and written consent was obtained from all the participants. Since 1999, NHANES has conducted annual surveys, and data are released every two years for public use. 

### 2.1. Study Design and Sample 

**Figure 1:**
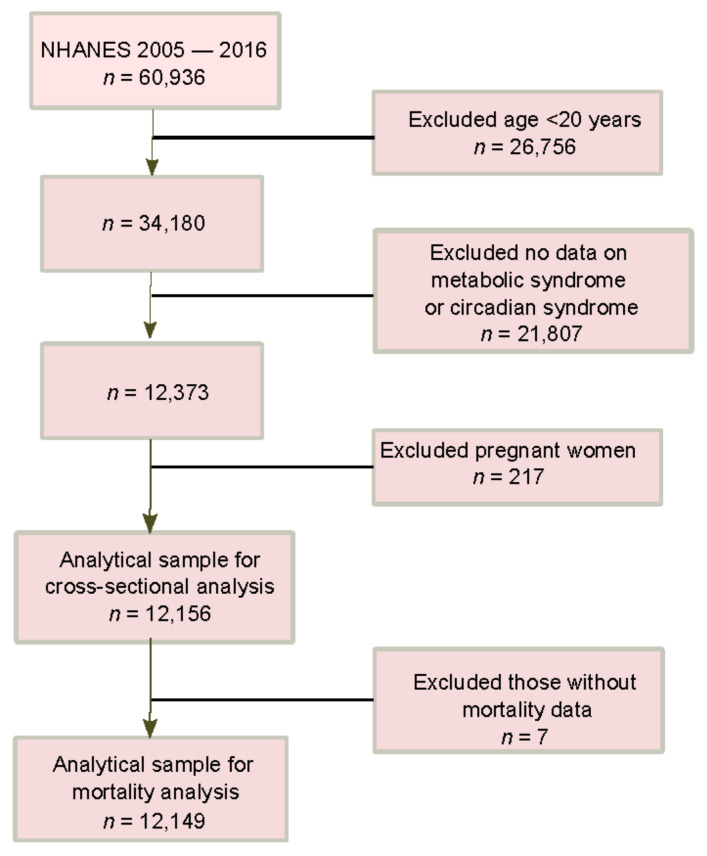
Sample flow chart. Abbreviations: NHANES, National Health and Nutrition Examination Survey.

In the current analysis, we used data from six survey cycles (2005–2006, 2007–2008, 2009–2010, 2011–2012, 2013–2014, and 2015–2016) with 12,156 participants (Figure 1). Participants below the age of 20 years were excluded from the study. NHANES has collected sociodemographic, lifestyle (including sleep), disease history, and health-related data that include clinical measures of blood pressure, fasting blood glucose, serum lipids, including triglycerides and high-density lipoprotein (HDL) cholesterol, and self-reported drug use for health conditions. Participants with missing values of components used to define MetS or CircS components were excluded from the study. The final analytical sample for the cross-sectional analysis included 12,156 participants (Figure 1), but seven individuals had missing data on mortality. 

### 2.2. Exposure measures: Metabolic Syndrome and Circadian Syndrome

Depression was measured using the Patient Health Questionnaire (PHQ-9). It is a nine-item screening instrument that asks questions about the frequency of depression symptoms over the past two weeks. Participants with a PHQ-9 score of ≥5 were defined as having depression symptoms. 

MetS was defined using the harmonized criteria proposed in the joint interim statement of the International Diabetes Federation Task Force on Epidemiology and Prevention, National Heart, Lung, and Blood Institute, American Heart Association, World Heart Federation, International Atherosclerosis Society, and International Association for the Study of Obesity [2] (Table 1). An individual was categorized as having MetS if he/she had ≥3 of the following metabolic disorder components: (1) elevated waist circumference (≥102 cm in men, ≥88 cm in women); (2) high blood pressure (Systolic ≥130 mm Hg and/or diastolic ≥85 mm Hg) or drug treatment for hypertension; (3) low HDL-cholesterol (<40 mg/dL in men and <50 mg/dL in women) or drug treatment for dyslipidemia; (4) high triglycerides (TG) (≥150 mg/dL) or drug treatment for dyslipidemia; or (5) elevated fasting glucose (≥100 mg/dL, drug treatment of elevated glucose is an alternate indicator). 

CircS was assessed based on seven components including self-reported short sleep (<6 h/day), depression symptoms and the five components used to define MetS. A cut-off for CircS was set as ≥4 components (Table 1). Based on MetS and CircS, a third variable was constructed for each individual with a possible value of: normal, MetS alone, CircS alone, and both MetS and CircS.

### 2.3. Outcome Measure: Self-Reported History of CVD and CVD Mortality

Participants were asked whether they had ever been diagnosed with congestive heart failure, coronary heart disease, angina, heart attack, or stroke by a doctor or other health professional. A positive answer to any of the conditions was defined as having prevalent CVD.

Mortality data were obtained via probabilistic matching to the death certificates from the National Death Index recorded up to 31 December 2019. In the analyses, the cause of death was coded according to the International Classification of Diseases, Tenth Revision (ICD-10). CVD mortality was defined based on ICD codes I00–I99. This method has been validated and widely used [15,16]. 

### 2.4. Covariates

The following self-reported variables were treated as covariates: age (years), sex, race (whites, blacks, Mexican Americans, other race), education level (recoded as lower than high school, graduated from high school or equivalent institution, any college, and college graduate or above), physical activity (based on the Global Physical Activity Questionnaire (GPAQ),the metabolic equivalent of task (MET) minutes was calculated and recoded into three levels: <600, 600–1200 and >1200 MET min/week), smoking status (non-smoker, ex-smoker and current smoker), and alcohol drinking. Poverty Income Ratio (PIR) was calculated by dividing the income of the family by the poverty threshold of the family and was categorized on three levels: <1.3 (low), 1.3–3.5 (moderate), and >3.5 (high) [17].

### 2.5. Statistical Analyses

Sex-specific analyses were conducted. Multivariable logistic regression or Cox regression analyses were used to examine the associations between MetS or CircS and prevalent CVD and CVD mortality, respectively. Survey weight was used in the multivariable model to account for the complex multistage probability sampling. Two multivariable logistic models were used: model 1, adjusted for age; and model 2, adjusted for age, education, race, income-poverty ratio, physical activity, smoking, and alcohol drinking. We calculated the positive predictive value (PPV) and negative predictive value (NPV) of MetS and CircS for prevalent CVD and CVD mortality. All analyses were conducted using STATA 17.0 (Stata Corporation, College Station, TX, USA). Statistical significance was considered when *p* < 0.05 (two sided). Stata codes for the analyses are available online (Supplementary Materials Code S1).

## 3. Results

The mean age of the participants was 49.8 years (Table 2). The unweighted prevalence of the CircS and MetS was 40.8% and 48.0%, respectively. In total, 259 (2.1%) and 1131 (9.3%) participants had either CircS or MetS alone and 4706 (38.7%) had both CircS and MetS. The CircS alone group had the highest prevalence of current smokers (34.4%) as compared with other groups. Women were more likely to have CircS alone than men. In contrast, men were more likely to have MetS alone than women. All participants in the CircS alone group had depression and short sleep duration by definition. Overall, short sleep (46.9%) was more common than depression symptoms (36.9%) among people with CircS.

Both MetS and CircS were associated with prevalent CVD. The unweighted prevalence of CVD was 20.4% among people with the CircS and 18.7% in those with the MetS, respectively, in comparison to 3.5% in the normal group (*p* < 0.001). In the fully adjusted multivariable logistic models for the CircS and MetS, the odds ratios (OR) for prevalent CVD were 2.92 (95% confidence interval (CI) 2.21–3.86) and 3.20 (2.38–4.30), respectively, in men (Table 3), and 3.27 (2.34–4.59) and 3.04 (2.15–4.30), respectively, in women. 

During a mean of 8.4 years of follow-up, there were 429 CVD deaths. The CVD mortality rate was 5.15 per 1000 person-years in people with CircS and 4.65 per 1000 person-years in those with MetS. The hazard ratio (HR, 95%CI) for CVD mortality was 1.20 (0.85–1.70) and 1.14 (0.77–1.71) in men, and 1.55 (1.12–2.13) and 1.36 (0.93–1.99) in women associated with CircS and MetS, respectively. Having both MetS and CircS was associated with more than a 54% increased risk of CVD mortality in women compared with women without either the MetS or CircS (Table 4). In women with CircS only, there was a significant increase in both prevalent CVD and CVD mortality. 

CircS had a higher PPV for prevalent CVD than MetS in both men (23.1% vs. 20.9%, respectively) and women (17.9% vs. 16.4%, respectively) (Supplementary Materials Table S1). CircS and MetS had a similar NPV for prevalent CVD in both sexes. However, CircS and MetS did not differ for the CVD mortality prediction as indicated by PPV.

## 4. Discussion

In this large national representative population-based study of the US adults, we found that the newly defined CircS was a better predictor for prevalent CVD than the MetS. Having both MetS and CircS was associated with a more than three-fold risk of CVD compared with people without either of these syndromes alone. Having CircS alone was significantly associated with prevalent CVD and CVD mortality in women. 

### Comparison with Other Studies

In this study, the MetS was associated with a tripled risk of prevalent CVD. The finding is in line with previous studies. In a meta-analysis of 87 studies, Mottillo et al. found that MetS had a relative risk of 2.35 (95%CI 2.02–2.73) for CVD [4]. This is the third study that has assessed the prevalence of CircS and the association between CircS and CVD. Two additional components, short sleep and depression, were added into the components of MetS to construct the CircS, which is also defined by more stringent criteria requiring ≥4 instead of ≥3 components. Indeed, using this new definition of CircS, 1131 (9.3%) participants (MetS alone) who were defined as having MetS in this study were found to be unqualified as regards having CircS (Table 2). Although it requires a higher stringency to define CircS, 259 (2.1%) additional participants (CircS alone) were identified, who were otherwise found to have no MetS. This reflects the additional value of the components of short sleep and depression added to define CircS. Existing evidence suggests that both short sleep and depression are associated with increased risk of CVD [18,19]. Using data from NHANES 2005–2018, Wang et al. found that the CircS was associated with stroke [20]. Consistent with our previous study in the Chinese population [12], the CircS was associated with CVD in the current study in the population of USA. Furthermore, the CircS had a better predictive value for prevalent CVD than the MetS as indicated by PPV.

An interesting finding of our study was that CircS alone in women was associated with a high likelihood of prevalent CVD and risk of CVD mortality, especially in those aged 40–59 years at baseline. It may be speculated that women with menopause, with a short sleep duration and depression are placed at higher risk of CVD. This emphasizes the importance to incorporate depression and short sleep into the MetS cluster that will construct the CircS. The difference between the current study in the US and the previous study in China is that the prevalence of CircS alone was lower in the US population (2.1%) than in China (4.6%). It means that in the US population, the majority of individuals with CircS had MetS, suggesting a more severe consequence of circadian disruption. 

The circadian system has been highlighted as a major regulator of almost every aspect of human health and metabolism [21]. The challenge of using this knowledge in clinical medicine is the difficulty of detecting and diagnosing circadian disruption. The concept of CircS was initially proposed in 2019 based on the finding that circadian disruption was linked to many modern chronic conditions including the MetS [11]. In 2017, the Nobel Prize for Physiology or Medicine was awarded to discoveries of molecular mechanisms controlling the circadian rhythm [22]. The link between disruption of circadian rhythms and MetS has been well-established [23]. It has been reported that CircS is a better substitute for the MetS in predicting CVD [12] as well as lower urinary tract symptoms [13] in the Chinese population. The circadian rhythm disruption could underline the common etiology of MetS and play a key pathological role in the newly defined CircS as well as in other modern diseases. In modern life, the extensive use of artificial light, exposure to low light levels indoors during the day, controlled ambient temperature and constant food availability, shift work, and jet travel over many different time zones, and even daylight saving time, can disrupt circadian rhythms [11,24,25,26]. All these modern lifestyle impacts have been shown to be associated with the components used to define CircS in the current study, as well as with CVD. For example, shift work and sleep disorders have been found to cause circadian misalignment. This in turn increases the risk of cardiovascular diseases [27]. Artificial light at night has adverse effects on psychological, cardiovascular and metabolic functions [28], and sleep deprivation increases the risk of type 2 diabetes and obesity [29]. Furthermore, in the Multi-Ethnic Study of Atherosclerosis, increased variability in sleep duration and timing (based on 7-day actigraphy measurements) was positively associated with metabolic abnormalities [30]. Both meal timing and quality affect sleep quality [31,32]. A causal link between circadian misalignment and metabolic homeostasis has been found in clinical laboratory research [33], and even a mild disturbance such as daylight saving time resulted in an increase in the occurrence of acute myocardial infarction, especially in the first week after the spring shift [26]. 

The limitation of this study is the lack of repeated measure of CircS and MetS and some of their potential covariates. Sleep duration in the present study was self-reported rather than objectively measured. Future studies should focus on measuring sleep patterns objectively using polysomnography [34,35] and measuring the circadian pattern of cortisol or of hormones involved in metabolic physiopathology (such as thyroid hormones [36]), which can objectively ascertain whether the chrono organization of the patients is actually altered or not. The cut-off used for depression symptoms based on PHQ-9 was 5 instead of 10. This is used for mild depressive symptoms, not for clinical depression. The advantage of the use of PHQ-9 is that it is relatively easy to use and takes less time. Non-alcoholic fatty liver disease (NAFLD) was part of the original proposed CircS [11]. In NHANES 1999–2012, the prevalence of NAFLD was 30% among adults aged 18 years and above [37]. In order to compare NHANES results with our previous study in China, we did not include NAFLD in the definition of CircS. The use of self-reported CVD is another limitation. However, self-reported CVD has been shown to be reliable to ascertain symptomatic non-fatal events [38,39]. Furthermore, CVD mortality was obtained via data linkage to the national mortality registry. Based on the components of MetS, we only added short sleep and depressive syndrome to define CircS. This could explain the lack of increased mortality of CircS as compared with MetS. The strength of the present study is the use of NHANES database, as it is representative of the US general population and has a large sample size. Our findings can be generalized to the US population but studies are needed to validate whether these can be generalized to other populations. The findings of this study confirm our previous study in China on the association between CircS and CVD risk. This is the first study to examine the association between CircS and CVD mortality. It supports the use of CircS in the context of the CVD prevention in the clinical settings, as the assessment of sleep duration and depression symptom is relatively easy and inexpensive based on the measure of MetS. In practice, the use of CircS will enable the identification of those with CircS alone as a high risk group for CVD prevention. However, more studies are needed to validate the findings. To date, this is the third study comparing the use of CircS and MetS in predicting health outcomes. 

## 5. Conclusions

In conclusion, based on data from NHANES 2005–2016, both the MetS and CircS can predict CVD in the US adult population. Furthermore, the use of CircS can better predict prevalent CVD. CircS and MetS did not differ for the CVD mortality prediction. The use of CircS, by adding short sleep and depression to the MetS and increasing the defining stringency, may afford both a cost- and health-effective approach to identify the risk populations in the prevention of CVD, especially in women. 

## Figures and Tables

**Table 1:** Definition of metabolic syndrome and circadian syndrome.

Measure	Categorical Cut Points	Included in MetS *	Included in CircS
Elevated waist circumference	≥102 cm in men, ≥88 in women	√	√
Elevated triglycerides (drug treatment for elevated triglycerides is an alternate indicator)	≥150 mg/dL (1.7 mmol/L)	√	√
Low HDL-C (drug treatment for reduced HDL-C is an alternate indicator)	<40 mg/dL (1.0 mmol/L) in men; <50 mg/dL (1.3 mmol/L) in women	√	√
Elevated blood pressure (antihypertensive drug treatment in a patient with a history of hypertension is an alternate indicator)	Systolic ≥130 mm Hg and/or diastolic ≥85 mm Hg	√	√
Elevated fasting glucose (drug treatment of elevated glucose is an alternate indicator)	≥100 mg/dL	√	√
Short sleep	≤6 h/day		√
Depression symptom	PHQ-9 score ≥5		√
Definition criteria		≥3 components	≥4 components

* MetS was defined based on reference [2]. √ means included. Abbreviations: MetS, Metabolic Syndrome; CircS, Circadian Syndrome; HDL-C, high-density lipoprotein cholesterol; PHQ-9, Patient Health Questionnaire.

**Table 2:** Sample characteristics by MetS and CircS status among adults aged above 20 years attending the US National Health and Nutrition Survey (NHANES 2005–2016, *n* = 12,156).

	Total	Normal	CircS Alone	MetS Alone	MetS and CircS	*p*-Value *
	*n* = 12,156	*n* = 6060 (49.9%)	*n* = 259 (2.1%)	*n* = 1131 (9.3%)	*n* = 4706 (38.7%)	
Age (years) (mean, SD)	49.8 (17.7)	42.5 (16.6)	47.6 (15.8)	54.0 (16.6)	58.3 (15.1)	<0.001
Age (years)						<0.001
20–39	32.6%	49.4%	34.0%	22.4%	13.4%	
40–59	33.6%	32.4%	41.7%	35.5%	34.1%	
60+	33.8%	18.2%	24.3%	42.1%	52.5%	
Sex						<0.001
Men	50.3%	50.8%	43.6%	55.1%	48.9%	
Women	49.7%	49.2%	56.4%	44.9%	51.1%	
Education						<0.001
<11 grade	25.3%	21.4%	33.2%	24.6%	30.1%	
High school	22.9%	21.1%	25.5%	24.0%	24.7%	
Some college	28.6%	28.9%	26.3%	28.0%	28.6%	
Higher than college	23.2%	28.6%	15.1%	23.3%	16.7%	
Race						<0.001
Non-Hispanic White	45.6%	43.4%	35.9%	49.7%	48.0%	
Non-Hispanic Black	19.4%	19.6%	30.1%	15.2%	19.6%	
Mexican American/Hispanic	15.7%	15.9%	15.8%	17.0%	15.2%	
Others	19.3%	21.1%	18.1%	18.1%	17.3%	
Income to poverty ratio						<0.001
<1.30	31.2%	29.5%	43.9%	27.3%	33.7%	
1.3–3.5	37.8%	37.1%	34.3%	37.1%	39.2%	
>3.5	31.0%	33.5%	21.8%	35.6%	27.1%	
Smoking						<0.001
Never	53.8%	58.1%	49.8%	53.3%	48.5%	
Former	25.1%	20.1%	15.8%	30.1%	30.9%	
Current smoker	21.1%	21.8%	34.4%	16.6%	20.5%	
Drinking						<0.001
No	18.0%	12.9%	22.8%	17.9%	24.5%	
Yes	68.0%	74.6%	64.1%	67.9%	59.8%	
Missing	13.9%	12.5%	13.1%	14.1%	15.7%	
Physical activity (METs minutes/week)						<0.001
<600	39.6%	32.3%	43.8%	40.2%	48.7%	
600–1200	11.4%	11.4%	12.0%	12.5%	11.2%	
≥1200	49.0%	56.3%	44.2%	47.3%	40.1%	
BMI (kg/m^2^) (mean, SD)	29.0 (6.7)	26.3 (5.5)	29.8 (7.0)	30.3 (6.1)	32.0 (6.8)	<0.001
CVD	10.9%	3.5%	9.3%	8.8%	21.0%	<0.001
Hypertension	36.3%	13.0%	32.4%	42.1%	65.1%	<0.001
Central obesity	56.5%	32.4%	64.1%	69.6%	84.0%	<0.001
Elevated glucose	53.3%	28.0%	40.2%	68.7%	83.0%	<0.001
Elevated triglycerides	42.1%	10.0%	21.2%	49.8%	82.8%	<0.001
Elevated blood pressure	48.6%	22.4%	47.9%	59.9%	79.7%	<0.001
Reduced HDL-C	44.4%	13.3%	26.6%	52.1%	83.6%	<0.001
Depression symptoms	23.5%	17.0%	100.0%	0.0%	33.4%	<0.001
Sleep ≤ 6 h/day	35.2%	32.2%	100.0%	0.0%	44.0%	<0.001
Circadian syndrome	40.8%	0.0%	100.0%	0.0%	100.0%	<0.001
Metabolic syndrome	48.0%	0.0%	0.0%	100.0%	100.0%	<0.001
Died during follow-up	11.3%	6.7%	10.0%	13.0%	17.0%	<0.001
Died from CVD during follow-up	3.5%	1.8%	2.7%	4.3%	5.7%	<0.001

* *p*-values were 2 sided and generated from analysis of variance for continuous variables and chi-square for categorical variables. Abbreviations: BMI, body mass index; CVD, cardiovascular disease; CircS, circadian syndrome; MetS, metabolic syndrome; SD, standard deviation; METs, metabolic equivalent of tasks.

**Table 3:** Association (odds ratio; 95% confidence interval) between metabolic syndrome (MetS) and circadian syndrome (CircS) with the prevalence of CVD and CVD mortality among adults attending the US National Health and Nutrition Survey (NHANES 2005–2016).

		MetS		CircS	
		Per one of MetS components (continuous)	MetS (yes vs. no)	Per one of CircS components (continuous)	CircS (yes vs. no)
Prevalent CVD					
Men (*n* = 6113)					
	Model 1 *	1.54 (1.39–1.71)	3.20 (2.38–4.30)	1.53 (1.41–1.66)	3.01 (2.28–3.97)
	Model 2 †	1.56 (1.40–1.72)	3.20 (2.38–4.30)	1.51 (1.39–1.64)	2.92 (2.21–3.86)
Women (*n* = 6043)					
	Model 1 *	1.57 (1.43–1.72)	3.62 (2.55–5.14)	1.59 (1.47–1.71)	4.02 (2.87–5.63)
	Model 2 †	1.49 (1.36–1.64)	3.04 (2.15–4.30)	1.50 (1.39–1.63)	3.27 (2.34–4.59)
CVD mortality					
Men (*n* = 6108)					
	Model 1 *	1.11 (0.99–1.25)	1.20 (0.81–1.77)	1.16 (1.04–1.29)	1.32 (0.93–1.88)
	Model 2 †	1.10 (0.97–1.24)	1.14 (0.77–1.71)	1.12 (1.00–1.25)	1.20 (0.85–1.70)
Women (*n* = 6041)					
	Model 1 *	1.16 (1.05–1.27)	1.52 (1.07–2.15)	1.23 (1.12–1.36)	1.76 (1.30–2.38)
	Model 2 †	1.12 (1.00–1.25)	1.36 (0.93–1.99)	1.19 (1.07–1.33)	1.55 (1.12–2.13)

Values are odds ratio (95%CI) derived from multivariable logistic regression models for prevalent CVD and hazard ratio (95%CI) from Cox regression for CVD mortality. * Model 1 adjusted for age. † Model 2 further adjusted for education, race, physical activity, smoking (never, former, current), and alcohol drinking (no, yes, missing). Abbreviations: CircS, circadian syndrome; MetS, metabolic syndrome; CI, confidence interval.

**Table 4:** Association between metabolic syndrome (MetS) and circadian syndrome (CircS) status and CVD among adults attending the US National Health and Nutrition Survey (NHANES 2005–2016).

			Health Status		
		Normal	CircS alone	MetS alone	CircS and MetS
Prevalent CVD					
Men (*n* = 6113)					
	Model 1 *	1.00	1.49 (0.62–3.56)	1.83 (1.16–2.90)	3.67 (2.69–5.02)
	Model 2 †	1.00	1.16 (0.48–2.79)	1.88 (1.18–2.98)	3.61 (2.65–4.94)
Women (*n* = 5908)					
	Model 1 *	1.00	4.15 (2.00–8.61)	1.65 (0.81–3.35)	4.64 (3.19–6.74)
	Model 2 †	1.00	2.60 (1.25–5.42)	1.54 (0.78–3.04)	3.75 (2.59–5.42)
CVD mortality					
Men (*n* = 6108)					
	Model 1 *	1.00	2.87 (0.69–11.89)	1.09 (0.64–1.87)	1.32 (0.89–1.95)
	Model 2 †	1.00	1.83 (0.43–7.75)	1.10 (0.63–1.92)	1.22 (0.82–1.80)
Women (*n* = 5906)					
	Model 1 *	1.00	3.16 (1.03–9.64)	1.03 (0.47–2.23)	1.74 (1.22–2.49)
	Model 2 †	1.00	2.52 (0.82–7.76)	1.01 (0.47–2.20)	1.54 (1.04–2.26)

Values are odds ratio (95%CI) derived from multivariable logistic regression models for prevalent CVD and hazard ratio (95%CI) from Cox regression for CVD mortality. * Model 1 adjusted for age † Model 2 further adjusted for education, race, physical activity, smoking (never, former, current), and alcohol drinking (no, yes, missing).

## Data Availability

The data used in the study are publicly available from the NHANES website.

## Supplementary Materials

The following supporting information can be downloaded at: https://www.mdpi.com/article/10.3390/nu14245317/s1, Table S1: Positive predictive value (PPV) (95%CI), negative predictive value (NPV) for metabolic syndrome and circadian syndrome in predicting prevalent CVD and CVD mortality among adults attending the US National Health and Nutrition Survey (NHANES 2005–2016, *n* = 12,156); Code S1: Stata code for analyses.
