# Supplementary material for: The Circadian Syndrome Is a Significant and Stronger Predictor for Cardiovascular Disease than the Metabolic Syndrome—The NHANES Survey during 2005–2016
Source: Nutrients. 2022 Dec 14;14(24):5317. doi: 10.3390/nu14245317 (PMC9785664; doi:10.3390/nu14245317)
Supplement: Supplementary file 1 [file nutrients-14-05317-s001.zip › nutrients-2042603-supplementary.pdf]

Table S1. Positive predictive value (PPV) (95%CI), negative predictive value (NPV) for metabolic syndrome and circadian syndrome in predicting prevalent CVD and CVD mortality among adults attending the US National Health and Nutrition Survey (NHANES 2005–2016, *n* = 12,156).

	**Men**		**Women**	
	**PPV (%)**	**NPV (%)**	**PPV (%)**	**NPV (%)**
Prevalent CVD				
MetS	20.9(19.4–22.4)	95.7(95.0–96.4)	16.4(15.1–17.8)	96.8(96.1–97.4)
CircS	23.1(21.4–24.8)	94.8(94.1–95.5)	17.9(16.4–19.4)	96.5(95.9–97.1)
CVD mortality				
MetS	6.2(5.3–7.1)	97.7(97.1–98.2)	4.7(3.93–5.50)	98.8(98.3–99.1)
CircS	6.3(5.4–7.4)	97.2(96.7–97.7)	4.8(3.98–5.68)	98.5(98.0–98.9)

Abbreviations: CircS, circadian syndrome; MetS, metabolic syndrome; CI, confidence interval; CVD, cardiovascular disease.
